# Dynamic Dystroglycan Complexes Mediate Cell Entry of Lassa Virus

**DOI:** 10.1128/mBio.02869-18

**Published:** 2019-03-26

**Authors:** Antonio Herrador, Chiara Fedeli, Emilia Radulovic, Kevin P. Campbell, Hector Moreno, Gisa Gerold, Stefan Kunz

**Affiliations:** a Institute of Microbiology, Lausanne University Hospital, Lausanne, Switzerland; b University of Lausanne, Lausanne, Switzerland; c Howard Hughes Medical Institute, Iowa City, Iowa, USA; d Senator Paul D. Wellstone Muscular Dystrophy Cooperative Research Center, The University of Iowa, Iowa City, Iowa, USA; e Department of Molecular Physiology and Biophysics, Roy J. and Lucille A. Carver College of Medicine, The University of Iowa, Iowa City, Iowa, USA; f Department of Neurology, Roy J. and Lucille A. Carver College of Medicine, The University of Iowa, Iowa City, Iowa, USA; g Department of Internal Medicine, Roy J. and Lucille A. Carver College of Medicine, The University of Iowa, Iowa City, Iowa, USA; h TWINCORE - Center for Experimental and Clinical Infection Research, Institute for Experimental Virology, Hannover, Germany; i Department of Clinical Microbiology, Umeå University, Umeå, Sweden; j Wallenberg Centre for Molecular Medicine (WCMM), Umeå University, Umeå, Sweden; University of Pittsburgh School of Medicine

**Keywords:** arenavirus, proteomics, tropism, viral entry, virus receptor

## Abstract

Recognition of functional receptors by viruses is a key determinant for their host range, tissue tropism, and disease potential. The highly pathogenic Lassa virus (LASV) currently represents one of the most important emerging pathogens. The major cellular receptor for LASV in human cells is the ubiquitously expressed and evolutionary highly conserved extracellular matrix receptor dystroglycan (DG). In the host, DG interacts with many cellular proteins in a tissue-specific manner. The resulting distinct supramolecular complexes likely represent the functional units for viral entry, and preexisting protein-protein interactions may critically influence DG’s function in productive viral entry. Using an unbiased shotgun proteomic approach, we define the largely unknown molecular composition of DG complexes present in highly susceptible epithelial cells that represent important targets for LASV during viral transmission. We further show that the specific composition of cellular DG complexes can affect DG’s function in receptor-mediated endocytosis of the virus. Under steady-state conditions, epithelial DG complexes underwent rapid turnover via an endocytic pathway that shared some characteristics with DG-mediated LASV entry. However, compared to steady-state uptake of DG, LASV entry via DG occurred faster and critically depended on additional signaling by receptor tyrosine kinases and the downstream effector p21-activating kinase. In sum, we show that the specific molecular composition of DG complexes in susceptible cells is a determinant for productive virus entry and that the pathogen can manipulate the existing DG-linked endocytic pathway. This highlights another level of complexity of virus-receptor interaction and provides possible cellular targets for therapeutic antiviral intervention.

## Abstract (precis)

Recognition of cellular receptors allows emerging viruses to break species barriers and is an important determinant for their disease potential. Many virus receptors have complex tissue-specific interactomes, and preexisting protein-protein interactions may influence their function. Combining shotgun proteomics with a biochemical approach, we characterize the molecular composition of the functional receptor complexes used by the highly pathogenic Lassa virus (LASV) to invade susceptible human cells. We show that the specific composition of the receptor complexes affects productive entry of the virus, providing proof-of-concept. In uninfected cells, these functional receptor complexes undergo dynamic turnover involving an endocytic pathway that shares some characteristics with viral entry. However, steady-state receptor uptake and virus endocytosis critically differ in kinetics and underlying signaling, indicating that the pathogen can manipulate the receptor complex according to its needs. Our study highlights a remarkable complexity of LASV-receptor interaction and identifies possible targets for therapeutic antiviral intervention.

## INTRODUCTION

The Old World arenavirus Lassa virus (LASV) causes a severe hemorrhagic fever with high mortality and affects large parts of Western Africa. The virus is carried by rodents of *Mastomys* species, and human infection occurs mainly via reservoir-to-human transmission (1–3). Due to the high case fatality rate, lack of a protective vaccine, and limited therapeutic options, LASV is considered one of the most important emerging pathogens (4, 5). Arenaviruses are enveloped negative-strand RNA viruses with a life cycle confined to the cytoplasm (6). The viral genome is comprised of a small (S) RNA segment that encodes the envelope glycoprotein precursor (GPC) and nucleoprotein (NP) and a large (L) segment encoding the matrix protein (Z) and the viral RNA-dependent RNA polymerase (L). The GPC precursor undergoes processing by cellular proteases to yield a stable signal peptide (SSP), the N-terminal GP1, and the transmembrane GP2 (7). The mature virion GP spike of arenaviruses is comprised of trimers of SSP/GP1/GP2 heterotrimers that represent the functional units of virus attachment and entry (7–9).

Human transmission of LASV occurs mainly via inhalation of aerosolized contaminated rodent excreta or by contaminated food (10). Following early viral multiplication in epithelial tissues, the virus can disseminate, resulting in severe systemic infection with high viral loads in serum and many organs (3). A highly predictive factor for disease outcome is early viral load, suggesting competition between viral multiplication and the patient’s immune response (11). The currently limited treatment options make the development of novel therapeutics against LASV an urgent need. Antiviral drugs capable of limiting viral spread may provide the patient’s immune system a window of opportunity to develop a protective response. Targeting viral entry appears therefore as a promising strategy for therapeutic intervention.

Binding of a virus to its cellular receptor(s) is the first and most fundamental step of every viral infection (12, 13). The major cellular receptor for Old World and clade C New World arenaviruses is the ubiquitously expressed extracellular matrix (ECM) receptor dystroglycan (DG) (14, 15). In the host cell, DG provides a molecular link between the ECM and the cytoskeleton and is crucial for normal physiology (16). Synthesized as a single precursor, DG undergoes autoprocessing, yielding the peripheral α-DG recognized by ECM proteins and the transmembrane β-DG anchored to the actin cytoskeleton. The biological function of α-DG critically depends on posttranslational modification by the glycosyltransferase like-acetylglucosaminyltransferase (LARGE) that attaches chains of [Xyl-α1-GlcA-3-β1-3] copolymers (17, 18) known as “matriglycan” that in turn are crucial for binding to ECM proteins and arenaviruses (19–25). Most mammalian cells express DG core protein, whereas the functional glycosylation of α-DG is under tight tissue-specific control, making DG a “tunable” receptor (19). Interestingly, in human patients and experimental infection, the cellular tropism of Old World arenaviruses does not always correlate with the functional glycosylation of DG (26, 27). Productive viral entry into cells lacking functional DG may be explained by the presence of alternative LASV receptors, including molecules of the Tyro3/Axl/Mer (TAM) and T-cell immunoglobulin and mucin receptor (TIM) families, or C-type lectins (28–31). However, some tissues, including skeletal muscle, express high levels of functional DG (14) but seem refractory to Old World arenavirus infection *in vivo* (26, 27). Combining arenavirus reverse genetics with an *ex vivo* primary tissue culture model, de la Torre and colleagues recently pinpointed viral entry as the major barrier for infection of differentiated human myotubes (32). This suggests that expression of functional DG *per se* is not sufficient for productive entry and that yet unknown factors may influence DG’s receptor function.

Some viruses use endocytic pathways that are prelinked to their receptors (13, 33). This is illustrated by cell entry of viruses via cellular cargo receptors linked to clathrin-mediated endocytosis, such as vesicular stomatitis virus (VSV) using low-density lipoprotein receptor (LDLR) (34) or clade B New World arenaviruses that enter via transferrin receptor-1 (TfR1) (35–37). Other viruses are capable of hijacking routes of endocytosis that are normally not associated with the receptor molecules (13, 33), e.g., adenovirus 2 (AdV2) and AdV5 that use CAR and αv integrins as receptors, but enter by distinct endocytic pathways (38, 39).

Extensive biochemical analysis revealed an unusually complex “interactome” of DG in muscle (16, 40, 41). The α/β-DG subunits anchor the complex in the membrane where α-DG undergoes high-affinity binding to ECM proteins. The cytosolic domain of β-DG binds to the large adaptor protein dystrophin that anchors the complex to the cortical actin cytoskeleton and recruits the scaffold proteins syntrophin and dystrobrevin. In skeletal and cardiac muscle, DG further associates with stoichiometric ratios of α-, β-, γ-, and δ-sarcoglycans (42, 43). This “canonical” α/β/γ/δ-sacroglycan complex associates with the transmembrane protein sarcospan and stabilizes the DG complex in the plasma membrane (42, 43). Mutations in α-, β-, γ-, and δ-sarcoglycans in humans manifest clinically as limb-girdle muscular dystrophies (LGMD) (42–44). Correct assembly and transport of the sarcoglycan complex in muscle require simultaneous expression of all four sarcoglycans (45). Deficiency in individual sarcoglycans disrupts the entire sarcoglycan complex with marked reduction of functionally glycosylated DG at the sarcolemma, resulting in muscular dystrophy and cardiomyopathy (46–49).

In nonmuscle tissues, cell-specific expression of DG-associated proteins results in considerable heterogeneity in the molecular composition of DG complexes (50). The specific composition of DG complexes present in cells targeted by LASV *in vivo* is currently largely unknown. We hypothesized that preexisting protein-protein interactions may affect DG’s viral receptor function. Using an unbiased proteomic approach, we define the DG complexes in highly susceptible human cells and provide the first evidence that their molecular composition can indeed affect viral entry. We further demonstrate that epithelial DG complexes are highly dynamic and linked to an endocytic pathway resembling receptor-mediated uptake. However, viral entry occurs with faster kinetics and requires additional signaling, suggesting that the pathogen can manipulate the existing DG-linked pathway.

## RESULTS

### Molecular composition of functional DG complexes in susceptible human cells.

**FIG 1:**
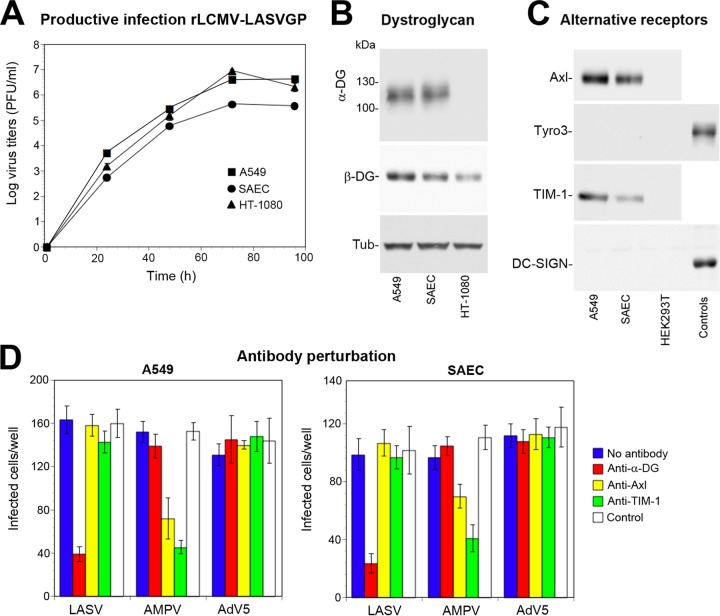
LASV candidate receptor expression and use in human respiratory epithelial cells. (A) Productive infection with rLCMV-LASVGP. A549 and HT-1080 cells and SAEC were seeded in 96-well tissue culture plates and infected with rLCMV-LASVGP (MOI of 0.1) for 1 h. At the indicated time points, cell culture supernatants were harvested, and infectious virus titers were determined by immunofocus assay (IFA) using MAb 113 to LCMV NP, combined with an Alexa Fluor 488-conjugated secondary antibody. Data are means ± standard deviations (error bars) (SD) from three experiments (*n* = 3). (B) Expression of functional DG in A549 cells and SAEC. Monolayers of A549 cells and SAEC were lysed, and cleared lysates were subjected to lectin affinity purification using wheat germ agglutinin (WGA). Eluted proteins were separated by SDS-PAGE and blotted onto nitrocellulose. Functional DG was detected with MAb IIH6 recognizing the matriglycan polymers on α-DG. The DG core protein was revealed with MAb 8D5 to β-DG. Tubulin (Tub) was detected in total cell lysates as a loading control. Primary antibodies were detected with HRP-conjugated secondary antibodies using enhanced chemiluminescence (ECL) for development. The human fibrosarcoma cell line HT-1080 that lacks functional DG was included as a negative control. (C) Detection of alternative LASV receptors in A549 cells and SAEC. Total cell protein was extracted from monolayers of A549 cells and SAEC. Axl, Tyro3, TIM-1, and DC-SIGN were detected with goat pAb anti-human Axl, MAb 96201 anti-human Tyro3, goat pAb anti-human TIM-1, and MAb 120507 anti-DC-SIGN, respectively. Human THP-1 monocytes and THP-1-derived immature dendritic cells were used as positive controls for Tyro3 and DC-SIGN, respectively (31). HEK293T cells were included as a negative control for Axl and TIM-1. Primary antibodies were detected as described above for panel B. The results from one representative experiment out of three independent experiments are shown. (D) Antibody perturbation of rLCMV-LASVGP entry in A549 cells and SAEC. Confluent monolayers of A549 cells and SAEC were blocked with MAb IIH6 to glycosylated α-DG (Anti-DG: 100 μg/ml), goat pAb anti-Axl (Anti-Axl: 20 μg/ml), goat pAb anti-TIM-1 (Anti-Axl: 20 μg/ml), and control antibodies (IgG + IgM) in the cold. Cells were then incubated with 300 PFU/well rLCMV-LASVGP (LASV), recombinant vesicular stomatitis virus (VSV) pseudotypes bearing the envelope GP of Amapari virus (AMPV), and recombinant adenovirus-5 expressing GFP (AdV5) 2 h in the cold in the presence of antibodies. Cells were washed and incubated with complete medium for 1 h. After 16 h of incubation in the presence of ammonium chloride, cells were fixed, and rLCMV-LASVGP infection was detected by IFA using MAb 113 to LCMV NP, combined with a Alexa Fluor 488-conjugated secondary antibody. Infection of AMPV pseudotypes and AdV5 was assessed by detection of GFP in direct fluorescence. Infection was quantified by counting infected cells per well considering cell doublets as single infection events. Data are means ± SD (*n* = 3).

Considering the data at hand, we hypothesized that cell-specific protein-protein interactions of DG may critically influence its function in viral entry. To test this possibility, we investigated the largely unknown molecular composition of DG complexes in highly susceptible human cells. For an unbiased analysis of DG complexes in susceptible cells, we employed shotgun proteomics with label-free quantification (LFQ). This approach uses affinity enrichment mass spectrometry (AE-MS) that avoids protein labeling and extensive purification to ensure minimal perturbation of the system and detection of weak interactions (51). For a suitable model, we chose respiratory epithelial cells that express functional DG (52–54) and are highly susceptible to LASV infection (3, 55). Since work with live LASV requires BSL4 facilities, we used a recombinant LCMV expressing the envelope GP of LASV (rLCMV-LASVGP) that is widely used to study LASV entry and tropism at BSL2 (30, 56, 57). Infection of primary human small airway epithelial cells (SAEC) and the immortalized human alveolar epithelial cell line A549 revealed comparable susceptibility to rLCMV-LASVGP (Fig. 1A). This correlated with the previously reported similar expression patterns of functional DG (Fig. 1B) and the alternative receptors Axl and TIM-1 (Fig. 1C) (30, 58). Antibody perturbation confirmed that productive entry of rLCMV-LASVGP into SAEC and A549 cells was mediated by DG, independently of Axl and TIM-1 (Fig. 1D) (28–30). The similar LASV receptor expression and specificity allowed using A549 cells as a suitable model for AE-MS studies that require large amounts of homogeneous material.

**FIG 2:**
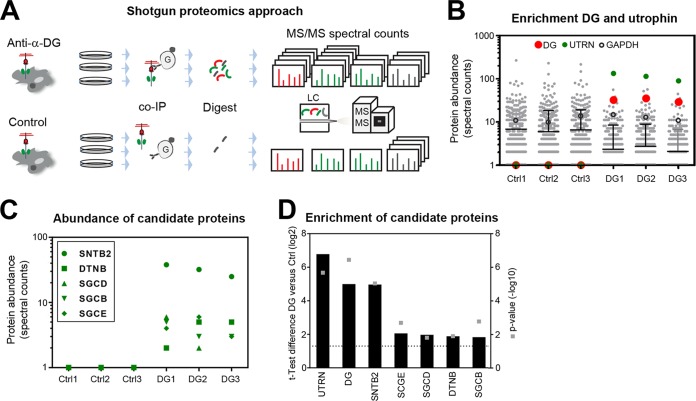
Shotgun proteomic analysis of DG complexes in A549 cells. (A) Schematic overview of the experimental setup used to identify DG-interacting proteins in A549 cells. A549 cells were lysed in the cold, lysates were incubated with blocked protein A/G Dynabeads, and precleared lysates were subjected to affinity enrichment using MAb VIA4 anti-matriglycan conjugated to protein A/G Dynabeads (DG) or anti-HA control Dynabeads (Ctrl). Enriched proteins were resolved by SDS-PAGE and tryptically digested, and the resulting peptides were identified by mass spectrometry. Relative protein abundance was determined by spectral counting. (B) Quality control for the DG affinity enrichment. Protein abundance of each identified protein quantified as spectral count in DG and Ctrl affinity enrichments from A549 cells. The bait DG (red) and utrophin (green) are highlighted. GAPDH (open black circles) served as a negative control. (C) Abundance of the identified DG complex proteins β2-syntrophin (SNTB2), β-dystrobrevin (DTNB), β-sarcoglycan (SGCB), δ-sarcoglycan (SGCD), and ε-sarcoglycan (SGCE) quantified as spectral counts. (D) Significant enrichment of candidate DG complex proteins from A549 cells was determined by nonparametric two-sample test correcting for multiple hypothesis testing (FDR of ≤0.05, s0 = 1; s0 is the minimal log_10_ fold change). The graph displays the difference of the median log_2_ spectral count of three biological replicates from DG-specific and isotype control purifications (left *y* axis, black bars) and the negative logarithmic *P* value (right *y* axis, gray squares). The dotted line shows the significance threshold of *P* < 0.05. See also Table 1.

To study the composition of the DG complex in A549 cells under steady-state conditions, we applied an AE-MS protocol we recently developed for analysis of virus receptor complexes (59). A challenge was that only a fraction of DG undergoes full functional glycosylation. To ensure specific AE-MS of functional DG complexes, we used monoclonal antibody (MAb) VIA4 that recognizes α-DG-linked matriglycan (60). Intact monolayers of A549 cells cultured under steady-state conditions in three biological replicates were extracted under mild nondenaturing conditions (Fig. 2A). Dystroglycan was subjected to AE-MS using MAb VIA4, including MAb anti-influenza hemagglutinin (HA) epitope as isotype control. Protein complexes were stabilized by chemical cross-linking with the thiol-cleavable reagent 3,3′-dithiobis(sulfosuccinimidyl propionate) (DTSSP). Proteins were separated by SDS-PAGE under reducing conditions and stained, and gels were cut into sections. After reduction, alkylation, and in-gel trypsin digestion, protein samples were separated via liquid chromatography (LC) and tandem MS analysis performed as detailed in Text S1 in the supplemental material. We observed a relatively high background in our controls due to the mild AE conditions (Table 1 and Fig. 2B). MS analysis quantified 1,169 proteins with a false discovery rate (FDR) of 0.1% on the peptide and 1.1% on the protein level (see Table S1 in the supplemental material). Stringent statistical analysis of our data sets revealed specific association of epithelial DG with 32 candidate proteins (FDR = 0.05%, s0 = 1). Major hits were the DG bait and the DG-binding adaptor protein utrophin that replaces dystrophin in nonmuscle tissues (Table 1 and Fig. 2B). Next, we searched data sets for other known DG-binding proteins identified in previous proteomic screens and other studies (40, 41). We detected the ECM proteins laminin-1, laminin-5, and agrin, the utrophin-binding partners β-dystrobrevin, and β2-syntrophin, and β-, δ-, and ε-sarcoglycan (Fig. 2C and Table 1).

**TABLE 1:** Proteins in the human epithelial cell α-DG complex^*a*^

Accession no.	−log *P* value	Difference (log_2_ DG − log_2_ Ctrl)	log_2_ spectral count					
			log_2_ CTRL1	log_2_ CTRL2	log_2_ CTRL3	log_2_ DG1	log_2_ DG2	log_2_ DG3
UTRO_HUMAN*	5,67461679	6,78010209	0	0	0	7,044394	6,820179	6,475733
DAG1_HUMAN*	6,44087927	4,99575472	0	0	0	5	5,129283	4,857981
SNTB2_HUMAN*	5,03351955	4,9639279	0	0	0	5,247928	5	4,643856
LAMA5_HUMAN	6,52146551	4,6211373	0	0	0	4,754888	4,584962	4,523562
AGRIN_HUMAN	4,16110658	3,73810649	0	0	0	3,584963	4,169925	3,459432
PDIA4_HUMAN	1,96151857	3,01842757	0	0	1,584962	3,584963	4,247928	2,807355
KDIS_HUMAN	1,39578508	2,9567883	0	0	0	4,169925	3,70044	1
UBR7_HUMAN	3,02039771	2,52832087	0	0	0	2,584963	3	2
AATC_HUMAN	2,6953076	2,49728433	0	0	0	2,321928	3,169925	2
GRP78_HUMAN	0,71406713	2,49657504	4,857981	0	4,70044	5,78136	5,807355	5,459432
MRP4_HUMAN	2,60125288	2,25162923	0	0	0	2,584963	2,584963	1,584962
RPIA_HUMAN	4,48726189	2,21461868	0	0	0	2,321928	2,321928	2
H4_HUMAN	2,86985745	2,19498758	1	1	1,584962	3,584963	3,584963	3
MDHC_HUMAN	2,41721832	2,07627289	0	0	1	2,321928	2,584963	2,321928
SGCE_HUMAN*	2,68002881	2,0566417	0	0	0	2	2,584963	1,584962
GPC1_HUMAN	2,68002881	2,0566417	0	0	0	1,584962	2,584963	2
SGCD_HUMAN*	1,79779233	1,96896354	0	0	0	2,584963	1	2,321928
4F2_HUMAN	0,69660978	1,92711989	0	0	3,584963	3,459432	3,584963	2,321928
SYHC_HUMAN (+1)	2,34472534	1,91829586	0	0	0	1,584962	2,584963	1,584962
DTNB_HUMAN*	1,88752281	1,88128535	0	0	0	1	2,321928	2,321928
ZA2G_HUMAN	0,98651543	1,83460859	3	1,584962	0	3,459432	3,169925	3,459432
SGCB_HUMAN*	2,76135407	1,83061767	0	0	0	2,321928	1,584962	1,584962
LAMB1_HUMAN	2,01115703	1,8170704	2	1,584962	1,584962	3,906891	3,906891	2,807355
LEG7_HUMAN	1,92392568	1,63563021	1	0	1	2	2,321928	2,584963
PSB4_HUMAN	1,83989995	1,63563017	0	0	1	2	2,321928	1,584962
PSA2_HUMAN	1,83989995	1,63563017	0	0	1	2	2,321928	1,584962
AATM_HUMAN	1,13901277	1,58496253	1	0	1,584962	2,584963	3,169925	1,584962
PLSL_HUMAN	1,35224274	1,52832087	0	0	0	1	2,584963	1
TPIS_HUMAN	2,18376989	1,44064271	1	1	1,584962	2,321928	2,584963	3
POF1B_HUMAN	1,7380005	1,38997499	1	0	0	1,584962	1,584962	2
RBBP4_HUMAN	2,68878129	1,38997499	0	0	0	1,584962	1,584962	1
CATA_HUMAN	2,08382834	1,31182003	2,321928	2,321928	3	4,087463	3,906891	3,584963
PSB2_HUMAN	1,49604625	1,07413081	1	1	1,584962	2	2,807355	2

a Proteins identified as α-DG interaction partners in A549 cells by affinity enrichment (AE) mass spectrometry and the spectral counts in each biological replicate are listed. Previously reported α-DG complex proteins are labeled with an asterisk. The enrichment of each protein by AE is shown as difference of median values of three biological replicates of DG-specific and control AE.

10.1128/mBio.02869-18.1 TEXT S1 Supplemental Materials and Methods. Detailed descriptions of proteomic analysis and virus internalization assays. Download Text S1, DOCX file, 0.05 MB. Copyright © 2019 Herrador et al. 2019 Herrador et al. This content is distributed under the terms of the Creative Commons Attribution 4.0 International license.

10.1128/mBio.02869-18.2 TABLE S1 Data analysis of the proteomic data sets. Download Table S1, XLSX file, 0.1 MB. Copyright © 2019 Herrador et al. 2019 Herrador et al. This content is distributed under the terms of the Creative Commons Attribution 4.0 International license.

**FIG 3:**
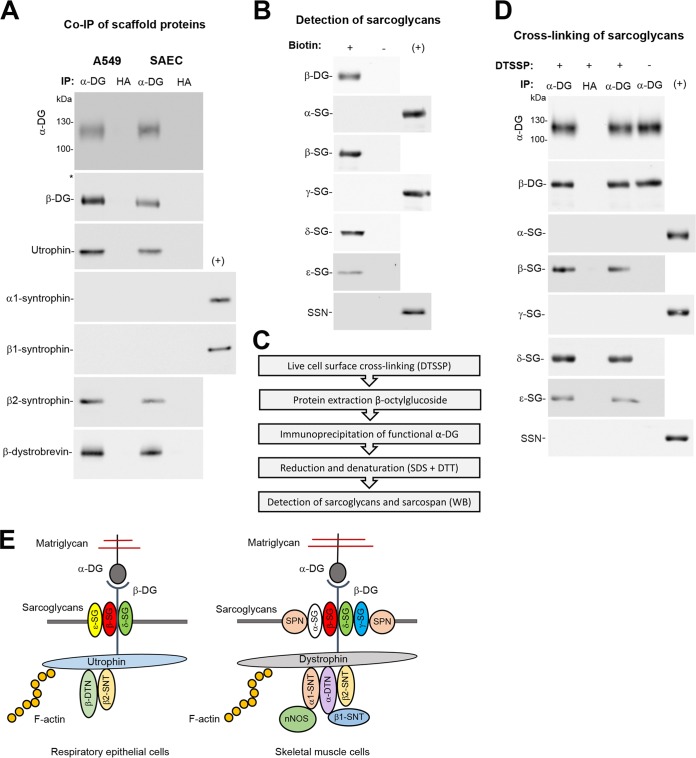
Validation of candidate DG-binding proteins. (A) Coimmunoprecipitation (co-IP) of DG-associated scaffold proteins. Monolayers of A549 and SAEC were lysed in cold Triton X-100 containing buffer, and cleared lysates were subjected to IP with MAb VIA4 matrix (α-DG) using MAb anti-HA matrix (HA) as a control. Immunocomplexes were probed in Western blots for functional α-DG (MAb IIH6), β-DG (MAb 8D9), utrophin (MAb 20C5), α1-syntrophin (goat pAb 5941), β1-syntrophin (rabbit pAb 98977), β2-syntrophin (MAb 1351), and β-dystrobrevin (rabbit pAb 152133). Primary rabbit and goat antibodies as well as MAb IIH6 (mouse IgM) were detected as described in the legend to Fig. 1B and C. For detection of mouse MAb IgG, HRP-conjugated mouse TrueBlot ULTRA secondary antibody was used as detailed in Materials and Methods. For a positive control (+), total lysates of differentiated human myotubes were included. One representative example out of three independent experiments is shown. (B) Detection of sarcoglycans at the surfaces of A549 cells. Intact monolayers of A549 cells were subjected to cell surface biotinylation using the membrane-impermeable reagent sulfo-NHS-X-biotin (+) or reaction buffer only (−). After reaction quenching, cells were lysed, and biotinylated proteins were precipitated with streptavidin agarose beads. Proteins were eluted and probed in Western blots for β-DG (MAb 8D9), α-sarcoglycan (SG) (rabbit pAb R98), β-sarcoglycan (MAb 5B1), γ-sarcoglycan (MAb 21B5), δ-sarcoglycan (rabbit pAb R214), ε-sarcoglycan (rabbit pAb 155651), and sarcospan (SSN) (rabbit MAb 186730), followed by detection as described above for panel A. The positive control (+) was differentiated human myotube lysate. One representative example of two independent experiments is shown. (C) Flow chart for the live cell surface cross-linking approach. For details, please see text. (D) Chemical cross-linking of DG with sarcoglycans at the cell surface. Live intact A549 monolayers were treated with the membrane-impermeable thiol-cleavable cross-linking reagent DTSSP (+) or reaction buffer only (−). After quenching, cells were lysed, and cleared lysates were subjected to IP with MAb VIA4 matrix (α-DG) or anti-HA matrix (HA) as described above for panel A. Eluted proteins were treated with DTT and analyzed in Western blot probing for functional α-DG, β-DG, the indicated sacroglycans, and sarcospan as described above for panel B. Please note that the blots for α- and β-DG (top) correspond each to 1% of the material, and the blots for the sacroglycans and sarcospan each correspond to 15% of the sample. One representative example of two independent experiments is shown. (E) Schema of a working model of the DG complex in A549 cells (this study) compared to the DG complex in skeletal muscle (based on published data [40, 41]). The α-DG-linked matriglycan sugar polymers and β-DG are indicated, as well as α-, β-, γ-, δ-, and ε-sarcoglycans (SG), sarcospan (SPN), α- and β-dystrobrevin (DTN), α1, β1, and β2-syntrophin (SNT), and nitric oxide synthase (nNOS).

To validate the association of epithelial DG with utrophin, β-dystrobrevin, and β2-syntrophin, monolayers of SAEC and A549 cells were extracted with cold Triton X-100, followed by immunoprecipitation (IP) with MAb VIA4. Western blotting revealed specific co-IP of DG with utrophin, β-dytrobrevin, and β2-syntrophin, but not α1- and β1-syntrophin in both cell types (Fig. 3A) consistent with our AE-MS (Fig. 2C and Table 1). To verify expression of candidate sarcoglycans, intact monolayers of A549 cells were subjected to cell surface biotinylation using sulfo-NHS-X-biotin as described previously (61). Biotinylated membrane proteins were separated by affinity purification using streptavidin beads. Western blot analysis confirmed the presence of β-, δ-, and ε-sarcoglycan, whereas α- and γ-sarcoglycan and sarcospan seemed absent (Fig. 3B). To detect a possible association of candidate sarcoglycans with DG in the plasma membrane, we performed chemical cross-linking with the membrane-impermeable reagent DTSSP on live intact A549 monolayers, followed by extraction with the detergent n-octyl β-D-glucoside that disrupts the sarcoglycan complex (62) and IP of functional α-DG. After stringent washing, immunocomplexes were eluted, followed by reduction to resolve cross-linked species. Western blotting revealed specific cross-linking of DG with β- and δ-sarcoglycan and to a lesser extent with ε-sarcoglycan (Fig. 3D). In sum, our biochemical validation confirmed the major candidate DG-binding proteins uncovered by our proteomic screen. The proposed molecular composition of epithelial DG complexes differs markedly from the well-described functional complexes in muscle (Fig. 3E).

### Candidate DG-binding proteins have no essential function in viral entry.

**FIG 4:**
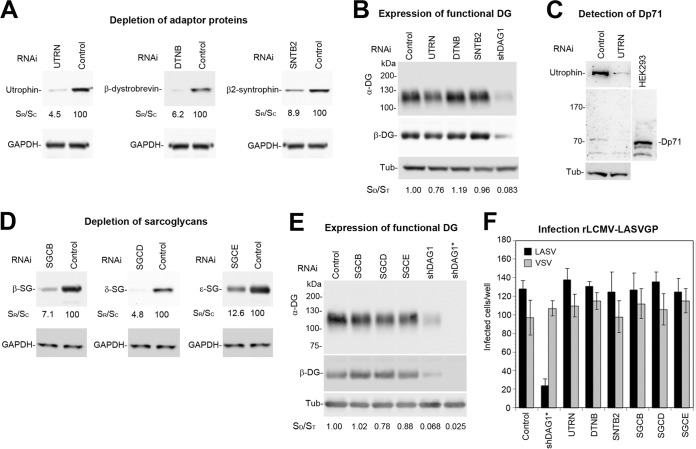
Candidate DG-binding proteins have no essential function in viral entry. (A) Depletion of candidate proteins by RNAi. A549 cells were transfected with siRNAs to utrophin (UTRN), β-dystrobrevin (DTNB), and β2-syntrophin (SBTB2) and scrambled control siRNAs (control) as detailed in Materials and Methods. After 72 h, expression of candidate proteins was detected in Western blots using GAPDH as a loading control. Efficiency of candidate protein depletion was assessed by densitometry, followed by calculation of the signal ratios of specific siRNA/control siRNA (S_R_/S_C_). (B) Detection of functional DG. A549 cells were subjected to RNAi (A), proteins were extracted, and WGA affinity purification was performed as described in the legend to Fig. 1B. Functional α-DG and β-DG were detected as described in the legend to Fig. 1B. For a positive control, we included A549 cells depleted for DG by shRNA (shDAG1). Tubulin (Tub) was detected in protein extracts to normalize for total protein content, and relative molecular masses are indicated. Changes in expression of functional α-DG were assessed by densitometry, followed by calculation of the signal ratios of α-DG/tubulin (S_D_/S_T_). One representative example of two independent experiments is shown. The mild reduction in functional α-DG in cells depleted for utrophin was consistently observed. (C) No compensatory upregulation of dystrophin Dp71. Total lysates of A549 cells transfected with siRNA to utrophin (UTRN) and control siRNA were probed with antibodies to utrophin and dystrophin, including HEK293 cells as a positive control. The position of Dp71 is indicated, and tubulin (Tub) was included as a positive control. Please note the lack of upregulation of Dp71 in utrophin-depleted cells. Blots were overexposed to detect residual amounts of dystrophin. (D) Depletion of sacroglycans. A549 cell underwent RNAi silencing with siRNAs to β-sarcoglycan (SGCB), δ-sarcoglycan (SGCD), and ε-sarcoglycan (SGCE) as in panel A, followed by verification of knockdown by Western blotting as described in the legend to Fig. 2B. Efficiency of depletion was assessed as described above for panel A, and GAPDH was included as loading control. (E) Expression of functional DG was validated as described above for panel B, including A549 cells depleted for DG by shRNA (shDAG1 and shDAG1*) as positive controls. (F) The known candidate DG-binding proteins are dispensable for viral entry. Cells depleted for candidate proteins as in panels A and D were infected with 300 PFU/well rLCMV-LASVGP (LASV) and rLCMV-VSVG (VSV), and infection was detected as described in the legend to Fig. 1D. Cells treated with scrambled siRNA and shDAG1* were included as negative and positive controls, respectively. Please note that cell entry of LASV into A549 cells largely depends on DG. Data are means ± SD (*n* = 3). One representative example of three independent experiments is shown.

To address a possible role for the DG-associated utrophin “scaffold” complex in functional receptor expression, we depleted utrophin, β-dystrobrevin, and β2-syntrophin from A549 cells by RNA interference (RNAi). Reduction of β-dystrobrevin and β2-syntrophin by >90% did not affect the levels of functional α-DG (Fig. 4B). Depletion of utrophin by >95% resulted in only mild reduction of functional α-DG expression (Fig. 4B), suggesting some sort of compensation. In previous studies, compensatory upregulation of the truncated dystrophin variant Dp71 in part rescued the utrophin null phenotype (50). To address this possibility, we probed lysates of utrophin-depleted and control cells for expression of Dp71 in Western blots. A549 cells expressed small amounts of Dp71 that did not change after utrophin depletion, excluding a compensatory mechanism (Fig. 4C). Our proteomic analysis further revealed the existence of a rudimentary β/δ/ε-sarcoglycan complex in epithelial cells (Fig. 3E). To assess a possible role in functional DG expression, we depleted β-, δ-, and ε-sacroglycan by RNAi. Significant depletion of the candidate β- and δ-sarcoglycans only mildly affected expression of functional DG (Fig. 4D and E). This seemed in sharp contrast to the well-documented situation in muscle tissue, where deficiency of α-, β-, or δ-sarcoglycan results in disruption of the entire sarcoglycan complex with marked reduction of functionally glycosylated DG and a dystrophic phenotype (46–49).

The only mildly altered expression of functional DG after depletion of candidate proteins did not automatically exclude a possible role for them in DG-mediated viral entry. As expected, depletion of the DG core protein in A549 cells specifically reduced infection with rLCMV-LASVGP, but not recombinant LCMV expressing the G protein of vesicular stomatitis virus (rLCMV-VSVG) that enters independently of DG (Fig. 4F). In contrast, depletion of the candidate DG-binding proteins by RNAi had no significant effect on productive entry of rLCMV-LASVGP, indicating that they have no essential function in viral entry (Fig. 4F).

### The molecular composition of the cellular DG complex can affect virus uptake.

**FIG 5:**
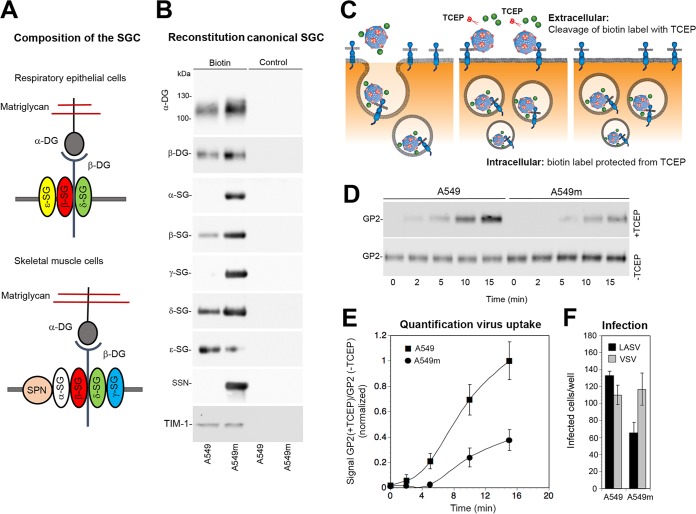
The molecular composition of the cellular DG complex can affect virus uptake. (A) Schematic representation of a working model of the DG-associated sarcoglycan complex in A549 cells and skeletal muscle. (B) Reconstitution of a muscle-type sarcoglycan complex in A549 cells. A549 cells were infected with recombinant AdV5 vectors expressing α-, β-, γ-, and δ-sarcoglycan (SG) and sarcospan (SSN) (A549m) or equal amounts of AdV5 expressing GFP (A549). After 48 h, cells were subjected to cell surface biotinylation with sulfo-NHS-X-biotin (biotin) or reaction buffer only (control) as described in the legend to Fig. 3B, and expression of the indicated proteins was assessed by Western blotting as described in the legend to Fig. 3. The mildly enhanced signal for functional α-DG and the weaker band for ε-sarcoglycan were consistently observed. One representative example of three independent experiments is shown. (C) Schematic of the virus internalization assay (for details, please see text). (D) Expression of a muscle-type sarcoglycan complement in A549m cells affects viral uptake. A549m and A549 cells were chilled on ice and incubated with biotin-S-S-labeled rLCMV-LASVGP (50 particles/cell) for 1 h in the cold. Unbound virus was removed, and cells were shifted to 37°C. After the indicated time points, cells were chilled on ice and treated with TCEP (+TCEP) or reaction buffer only (−TCEP). After quenching of residual TCEP, cells were lysed, viral GP was isolated by IP with MAb 83.6 to GP2. Biotinylated GP2 was detected with streptavidin-HRP in Western blots under nonreducing conditions using enhanced chemiluminescence (ECL). The top blot (+TCEP) was exposed for 5 min, and the bottom blot (−TCEP) was exposed for 30 s. One representative example of three independent experiments is shown. (E) Quantification of the three independent experiments (D). The intensity of GP2 signals in the presence or absence of TCEP was assessed by densitometry, followed by calculation of the signal ratios. For normalization, the value for the 15-min time point for A549 control cells was set at 1. Data are means ± SD (*n* = 3). (F) Infection of A549m and A549 cells. A549m and A549 cells in panel B were incubated with 300 PFU/well rLCMV-LASVGP (LASV) and rLCMV-VSVG (VSV), and infection was detected as described in the legend to Fig. 1D. Data are means ± SD (*n* = 3). One representative example of three independent experiments is shown.

In muscle, the “canonical” α/β/γ/δ-sacroglycan complex plus sacrospan is crucial for the stability of the functional DG complex in the plasma membrane (42, 43). In contrast, our data indicated that the truncated β/δ/ε-sarcoglycan complex detected in A549 cells seemed dispensable for the stabilization of functional DG, in line with distinct functions of sarcoglycans in muscle and epithelial tissue (Fig. 4). We therefore hypothesized that the specific composition of the sarcoglycan complex may affect DG turnover and hence viral entry. In muscle, deficiency in individual sarcoglycans disrupts assembly of the entire sarcoglycan complex and markedly reduces expression of functional DG, which is crucial for viral attachment (46–49). The very low levels of residual functional α-DG observed in δ-sarcoglycan-depleted myotubes prevented studies examining infection with rLCMV-LASVGP. To investigate possible effects of the specific composition of the sarcoglycan complex on DG-mediated viral entry in a well-controlled experimental system, we tried to reconstitute a “muscle-type” sarcoglycan complex in A549 cells and assessed the impact on viral entry (Fig. 5A). To this end, we overexpressed α-, β-, γ-, and δ-sarcoglycans plus sarcospan using adenoviral (AdV) vectors, resulting in A549m cells, as detailed in Materials and Methods. Control cells underwent infection with equal amounts of AdV expressing GFP. Cell surface biotinylation combined with Western blotting verified coexpression of α-, β-, γ-, and δ-sarcoglycans with sarcospan in the plasma membrane of A549m cells (Fig. 5B). Reconstitution of a canonical sarcoglycan complex in A549m cells resulted in mildly enhanced levels of functional DG at the plasma membrane, possibly due to stabilization of the complex (Fig. 5B). We further consistently observed reduced expression levels of ε-sarcoglycan in A549m cells, perhaps due to competition with overexpressed α-sarcoglycan (Fig. 5B). To discern effects on DG-mediated virus endocytosis from later steps of viral entry, we used a well-established virus internalization assay (63–65). In this assay, purified rLCMV-LASVGP was labeled with the thiol-cleavable reagent NHS-SS-biotin, resulting in a biotin label sensitive to reducing agents that does not affect infectivity. Treatment with the membrane-impermeable reducing agent Tris(2-carboxyethyl)phosphine (TCEP) in the cold specifically cleaves the biotin label from virus at the cell surface, but not from internalized virus particles (Fig. 5C). Monolayers of A549m and control A549 cells were chilled on ice and incubated with biotin-labeled rLCMV-LASVGP (50 particles/cell) for 1 h in the cold. Unbound virus was removed, and cells were rapidly shifted to 37°C. After the indicated time points, cells were chilled on ice and exposed to TCEP or reaction buffer in the cold. After quenching of residual TCEP, the cells were lysed and total viral GP2 was isolated by IP. Immunocomplexes were separated by SDS-PAGE under nonreducing conditions, and biotinylated GP2 was detected by Western blotting. We observed slightly higher virus binding to A549m cells in the absence of TCEP (Fig. 5D), consistent with the observed receptor expression (Fig. 5B). Treatment with TCEP revealed an apparent delay in virus internalization in A549m cells compared to control cells (Fig. 5D and E). Although our experimental setup is clearly artificial and has limitations, the data at hand provide the first evidence that the molecular composition of the functional DG complex can indeed affect uptake of viral particles. To validate this in the context of productive infection, A549m and A549 control cells were infected with rLCMV-LASVGP and rLCMV-VSVG. The latter infects cells independently of DG. Cells were fixed after 16 h, and productive infection was detected by IFA using an antibody to LCMV NP. We observed a consistent reduction of infection of A549m cells with rLCMV-LASVGP, but not rLCMV-VSVG (Fig. 5F), in line with delayed uptake of virus particles (Fig. 5D and E).

### Engagement by the virus affects the kinetics of DG uptake.

**FIG 6:**
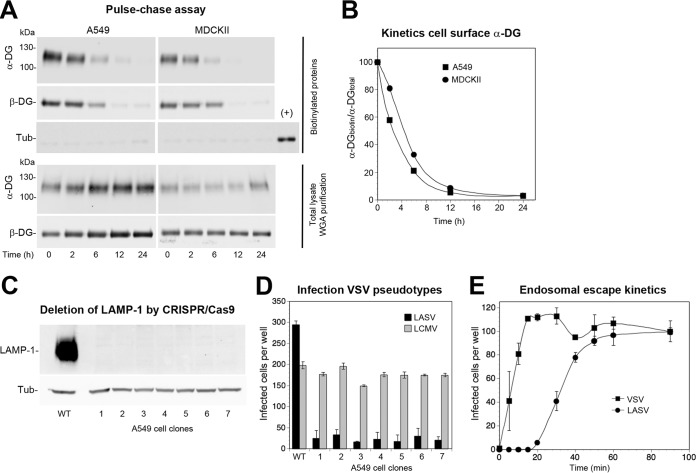
Steady-state DG uptake and viral entry kinetics. (A) Pulse-chase assay to assess steady-state DG turnover in uninfected cells. Intact monolayers of A549 and MDCKII cells were chilled on ice and subjected to cell surface biotinylation with sulfo-NHS-biotin, followed by reaction quenching in the cold. Cells were washed, prewarmed complete medium was added, and the temperature was shifted to 37°C to restore membrane fluidity. At the indicated time points, cells were chilled on ice, and lysis was performed with cold detergent buffer. Biotinylated proteins were precipitated from 80% of lysates with agarose beads, whereas 20% of lysates underwent WGA purification. As a specificity control for cell surface biotinylation, blots were probed for tubulin in the biotinylated fraction, including 1% of total lysate as a positive control (+). Please note the negligible signal for tubulin in the biotinylated protein fraction, validating the specificity of cell surface labeling. Functional α-DG and β-DG were detected in Western blots as described in the legend to Fig. 1B. One representative example of three independent experiments is shown. (B) Quantification of one out of three representative experiments by densitometry, followed by calculation of the signal ratios of biotinylated α-DG/total α-DG (α-DG_biotin_/α-DG_total_). (C) Generation of LAMP-1 null A549 cells. LAMP-1 was deleted from A549 cells by CRISPR/Cas9 as detailed in Materials and Methods and efficiency of depletion was assessed by Western blotting in seven individual clonal LAMP-1 null A549 cell lines using α-tubulin (Tub) as a loading control. (D) DG-dependent rVSVΔG-LASVGP infection of A549 cells depends on LAMP-1. The LAMP-1 null and control A549 cells (C) were infected with rVSV-LASVGP and rVSV-LCMVGP at 300 PFU/well for 1 h. Infection was assessed by direct fluorescence detection of the GFP reporter as described in the legend to Fig. 1D. Data are means plus SD (*n* = 3). (E) Kinetics of the endosomal escape of virus. rLCMV-LASVGP and rVSV-VSVG at 300 PFU/well were attached to monolayers of the indicated cells in the cold for 2 h in complete medium. Unbound virus was removed, and the cells were rapidly shifted to 37°C. At the indicated time points, 20 mM ammonium chloride was added and left throughout the experiment. After 16 h, infection was assessed by IFA as described in the legend to Fig. 1D. The means ± SD (*n* = 3) are given. Please note that the half-time of endosomal escape for rLCMV-LASVGP is <40 min. One representative example of three independent experiments is shown.

Many viruses use cellular receptors that are prelinked to pathways of endocytosis that are in turn involved in viral cell entry. For instance, the cargo receptors transferrin receptor 1 and low-density lipoprotein receptor used by many viruses are linked to clathrin-mediated endocytosis (13, 66, 67). Previous studies performed with murine mammary gland cells demonstrated that DG in the plasma membrane undergoes turnover with a half-life of circa 12 h (68), suggesting the existence of a DG-linked endocytic pathway. In a next step, we investigated the turnover of DG complexes in uninfected epithelial cell monolayers performing pulse-chase experiments. In addition to A549 cells, we included renal epithelial MDCKII cells that are highly susceptible to LASV (69). Live intact A549 and MDCKII monolayers were subjected to cell surface biotinylation with the membrane-impermeable reagent sulfo-NHS-X-biotin, applied in the cold to prevent receptor internalization. After reaction quenching, cells were rapidly shifted to 37°C to restore membrane fluidity. After the indicated time points, cells were lysed, and total protein was extracted. Biotinylated proteins were isolated, washed under stringent conditions, and analyzed by Western blotting. The pulse-chase assay revealed rapid turnover of α/β-DG in both cell types with half-lives of 4 to 6 h (Fig. 6A and B).

Next, we investigated whether the virus simply uses the existing endocytotic pathway prelinked to DG in a passive manner or whether engagement by the pathogen changes the dynamics of receptor uptake. After binding to DG at the plasma membrane, the virus-receptor complex is internalized, followed by delivery to acidified late endosomes. There, the virus undergoes a “receptor switch,” engaging the late endosomal resident membrane protein 1 (LAMP-1) that is crucial for fusion triggering (70). Since LAMP-1-positive late endosomes are also involved in degradation of membrane receptors like DG (71, 72), this allowed us to some extent to compare the kinetics of the two systems. To verify LAMP1 dependence of LASV entry in our system, we used CRISPR/Cas9 technology to delete LAMP-1 in A549 cells (Fig. 6C). LAMP-1 dependence of LASV entry was assessed by infection with recombinant VSV pseudotypes bearing either LASV GP or the GP of LCMV clone 13 that is LAMP-1 independent (70). In line with previous reports, infection with rVSV-LASVGP, but not rVSV-LCMVGP, critically depended on LAMP-1 (Fig. 6D). To assess the kinetics of late endosome virus escape, we determined the time required for rLCMV-LASVGP to become resistant to ammonium chloride. When added to cells, ammonium chloride raises the endosomal pH instantly and blocks further entry without cytotoxicity (73, 74). Virus was incubated with confluent A549 cells at low multiplicity (MOI of 0.01) in the cold, allowing receptor binding without internalization. Unbound virus was removed, and cells were shifted to 37°C. At the indicated time points, ammonium chloride was added. After 16 h, the cells were fixed, and productive infection was quantified by IFA. LAMP-1-dependent late endosomal viral escape occurred with a half time of <40 min (Fig. 6E), which was >5 times faster than steady-state DG turnover (Fig. 6B).

**FIG 7:**
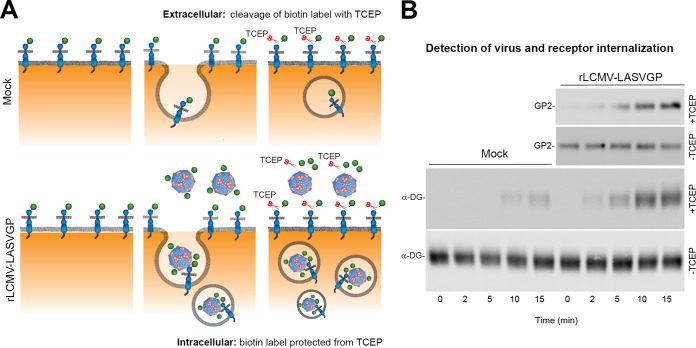
Engagement of rLCMV-LASVGP affects receptor internalization. (A) Schematic of the virus-induced receptor internalization assay. For details, please see text. (B) Virus engagement affects receptor internalization. Monolayers of A549 cells were subjected to cell surface biotinylation with the thiol-cleavable membrane-impermeable reagent sulfo-NHS-SS-biotin in the cold. Labeled cells were then incubated with biotin-SS-labeled rLCMV-LASVGP (50 particles/cell) or a mock virus preparation for 1 h in the cold as described in the legend to Fig. 5D, followed by removal of unbound virus. Cells were rapidly shifted to 37°C at the indicated time points, chilled on ice, and treated with TCEP (+TCEP) or reaction buffer only (−TCEP). Residual TCEP was carefully quenched, and cells were lysed. Cleared lysates were subjected to IP with MAb VIA4 matrix (α-DG) or MAb 83.6 to GP2 as indicated. Biotinylated α-DG and LASV GP2 were detected with streptavidin-HRP in Western blots under nonreducing conditions using ECL. The anti-GP2 blots in the presence and absence of TCEP were exposed for 2 min and 10 s, respectively. The anti-α-DG blot with TCEP was exposed for 5 min, and the anti-α-DG blot without TCEP was exposed for 30 s. One representative example of three independent experiments is shown.

The pulse-chase data and the endosomal escape analysis suggested differences in the kinetics of steady-state receptor uptake and viral entry (Fig. 6). However, a direct comparison of the data was not possible. Previous studies demonstrated that LASV binds functional DG with high affinity (14, 25, 75) and that an acidic pH of 5.5 is required to dissociate the virus from DG (70), suggesting internalization of the receptor complex during the entry process. To study possible effects of virus engagement on receptor uptake kinetics, we combined our conventional virus internalization assay (Fig. 5C) with labeling of cell surface DG with thiol-sensitive biotin. This new assay format allowed simultaneous monitoring of virus uptake and virus-induced receptor internalization (Fig. 7A). Live monolayers of A549 cells were subjected to cell surface biotinylation with the membrane-impermeable thiol-cleavable reagent sulfo-NHS-SS-biotin in the cold. The NHS moiety of the labeling reagent specifically reacts with primary amino groups present in the DG core protein and does not affect the integrity of the matriglycan sugars that represent the actual virus-binding site. After cell surface biotinylation, cells were subjected to virus internalization assay using purified rLCMV-LASVGP labeled with NHS-SS-biotin as described above. For a control, an unlabeled “mock” virus preparation obtained from uninfected cells was included. After the indicated time points, cells were chilled and treated with TCEP or reaction buffer in the cold. After careful quenching of TCEP, cells were lysed, and cleared lysates were subjected to IP using MAb 83.6 to LASV GP2 and MAb VIA4 to functional α-DG. Biotinylated GP2 and α-DG were detected in Western blots under nonreducing conditions using streptavidin conjugated to HRP. We detected weak signals for internalized receptor in the absence of virus after 10 to 15 min in cells treated with TCEP (Fig. 7B). This background level of receptor internalization was expected based on the steady-state uptake revealed by our pulse-chase assay (Fig. 6A and B). Addition of virus consistently resulted in detection of higher levels of biotinylated functional α-DG in specimens treated with TCEP (Fig. 7B), suggesting that engagement of virus could affect dynamics of receptor endocytosis.

### Differential signaling is required for steady-state DG turnover and DG-mediated viral entry.

**FIG 8:**
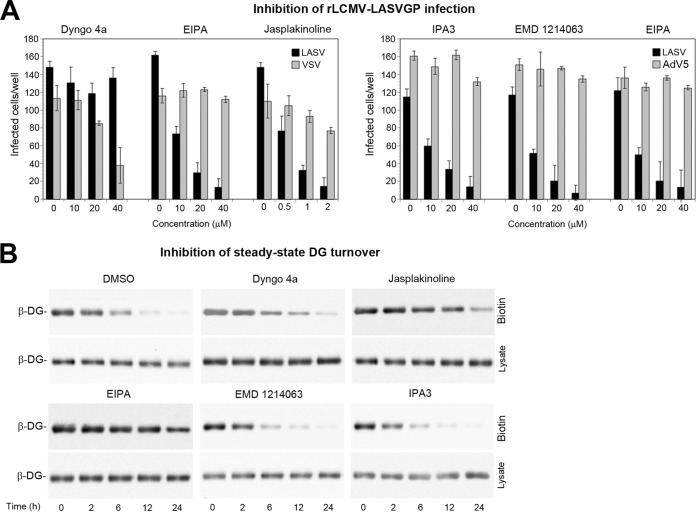
Differential signaling is required for steady-state DG turnover and DG-mediated viral entry. (A) Inhibition of rLCMV-LASVGP infection with selected inhibitors. A549 cells were pretreated with the Dyngo 4a, EIPA, jasplakinoline, IPA3, and EMD 1214063 at the indicated concentrations for 30 min, followed by infection with the rLCMV-LASVGP (LASV), rLCMV-VSVG (VSV), or AdV5 expressing GFP (AdV5) at 200 PFU/well in the presence of drug. After 1 h, cells were washed three times with medium containing 20 mM ammonium chloride, followed by 16 h of incubation in the presence of the lysosomotropic agent. Infection of rLCMV-LASVGP and rLCMV-VSVG was detected by IFA and AdV5-GFP by direct fluorescence as described in the legend to Fig. 1D. Data are means ± SD (*n* = 3). (B) Pulse-chase assay of cell surface DG in the presence of inhibitors. Intact monolayers of A549 cells were pretreated with the indicated drugs (Dyngo 4A, EIPA, EMD 1214063, and IPA3 [40 µM each] and jasplakinoline [2 µM]), chilled on ice, and subjected to cell surface biotinylation with NHS-X-biotin as described in the legend to Fig. 6A. Biotinylated proteins were precipitated from 90% of lysates with agarose beads, whereas 10% of lysates underwent total protein extraction. Beta-DG was detected in Western blots as described in the legend to Fig. 1B. One representative example of three independent experiments is shown.

Engagement of cellular receptors by many viruses induces signaling that can serve as a “knock on the door,” facilitating subsequent steps of entry (12). Recent studies demonstrated that DG-mediated cell entry of LASV involves an endocytotic pathway related to macropinocytosis, characterized by dynamin independence, and dependence on sodium proton exchangers (NHE), actin, and p21-activating kinase 1 (PAK1) (58, 76). Productive LASV entry into human epithelial cells further required signaling by receptor tyrosine kinases, in particular hepatocyte growth factor receptor (HGFR) (58). We therefore compared the sensitivity of viral entry and steady-state DG turnover to a panel of “diagnostic” inhibitors, including the dynamin inhibitor Dyngo 4a, jasplakinoline that prevents actin depolymerization, the NHE inhibitor 5-N-ethyl-N-isopropyl amiloride (EIPA), the HGFR inhibitor EMD 1214063, and the PAK1 inhibitor IPA3. Consistent with published reports, EIPA, jasplakinoline, EMD 1214063, and IPA3, but not Dyngo 4a, reduced rLCMV-LASVGP infection in a dose-dependent manner (Fig. 8A). To investigate effects on steady-state turnover of DG, monolayers of A549 cells were pretreated with drugs at concentrations that blocked >90% of viral infection (Fig. 8B), followed by pulse-chase assay in the presence of drug. Similar to viral entry, steady-state turnover of DG was independent of dynamin but showed some sensitivity to inhibitors of NHE and actin (Fig. 8B). In contrast to virus entry, inhibitors of HGFR and PAK1 only mildly affected the steady-state turnover of DG in uninfected cells (Fig. 8B). This suggested that the pathogen requires additional signals for productive entry.

## DISCUSSION

The interaction of a virus with its cellular receptor(s) represents a key determinant for species and tissue tropism of a virus and is of particular relevance in the context of emerging human pathogens like arenaviruses. The highly pathogenic LASV represents a particularly complex paradigm for virus-receptor interaction with important consequences for viral transmission and disease potential. A large body of evidence supports the notion that DG is a “tunable” receptor (19), whose virus-binding affinity is strictly dependent on its complex glycosylation (20). However, clinical and experimental data revealed that expression of functionally glycosylated DG *per se* is not sufficient for productive infection (3, 26, 27). We therefore hypothesized that the capacity of functionally glycosylated DG to serve as a virus receptor may be influenced by its complex tissue-specific protein-protein interactions. Specific DG-binding proteins expressed in susceptible cells may promote DG dynamics and facilitate virus entry, fulfilling the operational definition of auxiliary viral “entry factors.” On the other hand, refractory tissues may express DG-binding proteins that counteract its function in viral entry, e.g., by stabilization of the DG complex in the membrane and/or sorting into specific microdomains that do not allow productive viral entry.

Outside of muscle, tissue-specific expression of DG-binding proteins results in a high degree of heterogeneity of DG complexes and their molecular composition in relevant LASV target cells is not defined well (50). Early studies on the biochemical composition of DG complexes in adult lung tissue revealed the existence of two distinct DG complexes (52), one associated with smooth muscle comprised of β/δ/ε-sarcoglycan plus sarcospan, and an epithelial DG lacking a functional sarcoglycan complex. Extending these studies, we performed an unbiased shotgun proteomic screen for DG-binding proteins in homogeneous cultures of immortalized human lung epithelial cells. Stringent analysis of our data sets revealed specific association of epithelial DG with 32 candidate proteins, including the known DG-binding partners utrophin, β-dystrobrevin, β2-syntrophin, and β-, δ-, and ε-sarcoglycan. Combining co-IP and chemical cross-linking, we were able to verify the known DG-binding proteins among the top hits and propose a working model for the respiratory epithelial DG complex (Fig. 3E). Using RNAi silencing, we assessed the roles of the known candidate DG-binding proteins in functional receptor expression. Depletion of utrophin only mildly affected expression of functional DG, which seems consistent with the phenotype of utrophin null mice that have no morphological defects in epithelia (77). Depletion of β-dystrobrevin did not affect functional DG in our system, in line with the observation that β-dystrobrevin null mice show no obvious alterations of the DG complex in the lung (78). Out of the five known syntrophins, epithelial DG associated only with the β2-isoform that binds utrophin (79). Mice lacking β2-syntrophin have no obvious phenotype, suggesting functional redundancy (80), in line with the results of our study.

Our proteomic screen complemented by chemical cross-linking on intact cells suggested a transient interaction of β-, δ-, and ε-sarcoglycans with DG in epithelial cells. This was consistent with the previous observation that β- and δ-sarcoglycan can assemble a “core” complex in the absence of other sarcoglycans (81–83). The absence of sarcospan and perhaps other associated proteins may explain the lower stability of the β/δ/ε-sarcoglycan complex in A549 cells. Moreover, RNAi silencing of candidate sarcoglycans hardly affected functional DG expression, which is in contrast to the severe impact of β- and δ-sarcoglycan deficiencies in muscle (46, 49). Mice deficient in β-sarcoglycan lack the entire canonical sarcoglycan complex and show dramatically reduced functional DG in skeletal and heart muscle, resulting in severe muscular dystrophy and cardiomyopathy (49). The BIO 14.6 dystrophic hamster, a classical model for human LGMD, is deficient in δ-sarcoglycan, lacks the entire sarcoglycan complex and shows markedly reduced functional DG in skeletal muscle (46, 47). Therapeutic delivery of the δ-sarcoglycan gene rescues functional DG expression and ameliorates the disease phenotype (47). In sum, our data revealed expression of a restricted subset of sarcoglycans in epithelial cells that apparently fail to assemble into a stable functional complex, in line with the original report (52).

Depletion of DG-associated candidate proteins in our epithelial cells did not significantly affect viral entry, excluding significant roles as entry or restriction factors. Previous studies demonstrated that virus engagement of DG induces rapid tyrosine phosphorylation of a PXXY motif at the C terminus of β-DG, resulting in dissociation of DG from utrophin (65). Together with the data presented here, this suggests that virus-induced receptor clustering and signaling may actively uncouple the virus-receptor complex from the utrophin-based scaffold, making these proteins dispensable for viral entry. Since none of the candidate DG-binding proteins facilitated viral entry, we tested whether components that are normally absent from epithelial cells may negatively affect DG’s viral receptor function. Reconstitution of a muscle-type sarcoglycan complex in A549 cells resulted in mildly enhanced virus-cell binding but delayed internalization and overall made cells less susceptible to infection. This suggests that the specific molecular composition of the cellular DG complex may indeed affect productive viral entry.

Turnover of many membrane receptors, including DG, involves internalization by endocytosis, followed by delivery to late endosomes/lysosomes, where degradation takes place (71, 72). This pathway closely resembles the mechanism of LASV entry that critically depends on the late endosomal protein LAMP-1, making it a late-penetrating virus (70, 84). In the respiratory and renal epithelial cells used in our present study, DG turnover occurred with an estimated half-life of 4 to 6 h. In comparison, the LAMP-1-dependent late endosomal viral escape occurred with a half time of <40 min. This was >5-fold faster than the steady-state receptor turnover. Since a direct comparison of pulse-chase assay data with the viral entry studies was not possible, we combined the classical virus uptake assay with cell surface biotinylation of DG to monitor virus-induced receptor internalization. This assay format revealed that engagement of virus can accelerate receptor uptake. Comparing DG-mediated LASV entry and steady-state DG turnover further indicated that both are dynamin independent and require to some extent NHE and actin, suggesting some similarities in the uptake pathways. In contrast to steady-state DG turnover, viral entry critically depended on signaling through HGFR and PAK1, indicating that the pathogen requires additional signals for productive entry. We are currently investigating whether the virus actively induces HGFR and PAK1 signaling or whether these kinases provide permissive signals. In a follow-up to the present study, we are also investigating the roles of other, previously unknown candidate DG-binding partners uncovered in our shotgun proteomic screen and their possible link to RTK and PAK1 signaling. The specific role of HGFR and PAK1 in viral entry but not in normal DG trafficking make both kinases promising targets for therapeutic intervention, opening the possibility of repurposing some of the inhibitors currently evaluated in the clinic against other human diseases. In sum, we show that the specific molecular composition of dynamic DG complexes in susceptible cells favors virus entry and that the pathogen can manipulate the existing DG-linked pathway, highlighting another level of complexity of virus-receptor interaction.

## MATERIALS AND METHODS

### Antibodies and reagents.

Monoclonal antibodies 83.6 to LASV GP2 (85), 113 anti-LCMV NP (86), VI4A, and IIH6 to matriglycan have been described previously (60). MAb 8D5 anti-β-DG was from Novocastra. Polyclonal goat anti-human Axl, MAb 96201 anti-Tyro3, MAb 120507 anti-DC-SIGN, goat polyclonal anti-TIM-1 (AF1750), and MAb anti-TIM-1 (MAB1750) were from R&D Systems. Antibodies to utrophin MAb 20C5, α1-syntrophin goat polyclonal antibody (pAb) 5941, β1-syntrophin rabbit pAb 98977, β-dystrobrevin rabbit pAb 152133, MAb ab54803 anti-Axl, ε-sarcoglycan rabbit pAb 155651, and sarcospan rabbit MAb 186730 were from Abcam. Anti-β2-syntrophin MAb 1351 was from Thermo Fisher. Antibodies to α-sarcoglycan rabbit pAb R98, β-sarcoglycan MAb 5B1, γ-sarcoglycan MAb 21B5, δ-sarcoglycan rabbit pAb R214 were from the Howard Hughes Medical Institute, University of Iowa. MAb sc-20011 anti-LAMP1 was from Santa Cruz Biotechnology. Mouse MAb B-5-1-2 anti-α-tubulin was from Sigma-Aldrich. Horseradish peroxidase (HRP)-conjugated polyclonal goat anti-mouse IgG, goat anti-mouse IgM, and rabbit and anti-goat IgG were from Dako. FITC-conjugated goat anti-mouse IgG was from Jackson Immuno Research. The nuclear dye 4',6-diamidino-2-phenylindole (DAPI) was from Molecular Probes (Eugene, OR). Inhibitors used were Dyngo 4a (Ascent Scientific), EMD 1214063 (Selleckchem), 5-(N-ethyl-N-isopropyl) amiloride (EIPA), IPA3, jasplakinolide, and ammonium chloride (Sigma).

### Cell culture and viruses.

Human lung epithelial cells A549, the canine renal epithelial cell line MDCKII, human fibrosarcoma HT-1080 were originally obtained from ATCC and cultured in DMEM, 10% (vol/vol) FBS, supplemented with glutamine and penicillin/streptomycin. Primary human small airway epithelial cells (SAEC) (CC-2547) and primary human skeletal muscle myoblasts (HSMM) (CC-2580) were purchased from Clonetics/Lonza and cultured following the manufacturer’s protocol (32, 58). rLCMV-LASVGP and rLCMV-VSVG have been described previously (37, 87). Recombinant VSV pseudotypes bearing the envelope GPs of LASV Josiah, LCMV clone 13, AMPV, and VSV were generated as reported earlier (75, 88). Recombinant human AdV-5 expressing α-, β-, γ-, δ-sarcoglycan, sarcospan, and GFP were provided by the University of Iowa Viral Vector Core (http://www.medicine.uiowa.edu/vectorcore).

### Proteomic analysis.

For a detailed description of proteomic analysis, see Text S1 in the supplemental material.

### Chemical cross-linking.

A549 cells were grown in a confluent monolayer in 10-cm cell culture dishes and chilled on ice for 10 min. Cells were washed with cold PBS and subjected to cell surface cross-linking with 1 mM DTSSP (Pierce) in cold HBSS two times for 20 min each time at 4°C. The reaction was quenched for 15 min with cold 50 mM glycine, 150 mM NaCl, 1 mM MgCl_2_, 0.1 mM CaCl_2_ (pH 8.0), cells were briefly rinsed with cold PBS, and lysis buffer (1% [wt/vol] β-n-octyl-glucoside, 0.1% [wt/vol] SDS, 50 mM Tris-HCl [pH 8.0], 150 mM NaCl, 1 mM PMSF, protease inhibitor cOmplete [Roche]) was added.

### Cell surface biotinylation and pulse-chase assay.

Cells grown in confluent monolayers in six-well plates were washed with cold PBS and subjected to cell surface biotinylation with 1 mM EZ-Link-Sulfo-NHS-biotin (Thermo Scientific) in cold PBS two times for 20 min each time at 4°C. The reaction was quenched for 15 min with cold 50 mM glycine, 150 mM NaCl, 1 mM MgCl_2_, 0.1 mM CaCl_2_ (pH 8.0), prewarmed medium was added, and cells were shifted to 37°C. At the indicated time points, cells were chilled on ice, briefly rinsed with cold PBS, and lysis buffer (1% [wt/vol] Triton X-100, 1% [wt/vol] CHAPS, 0.1% [wt/vol] SDS, 50 mM Tris-HCl [pH 8.0], 150 mM NaCl, 1 mM PMSF, protease inhibitor cOmplete) was added. Lysates were cleared by centrifugation, and biotinylated proteins were precipitated from lysate with streptavidin agarose (Sigma) as described previously (61). Agarose beads were eluted by boiling in SDS-PAGE buffer, and the biotinylated proteins and total cell lysates were subjected to Western blot analysis.

### Immunoprecipitation.

For immunoprecipitation (IP), MAb VIA4 was coupled to cyanogen bromide-activated Sepharose (Sigma) and washed with 0.1 M glycine (pH 2.80), 150 mM NaCl followed by 50 mM Tris-HCl, 500 mM NaCl (pH 8.0). SAEC were cultivated in 10-cm dishes at 8 × 10^5^ cells per dish for 3 days, and A549 cells were cultivated in 10-cm dishes at 8 × 10^5^ cells per dish for 2 days. Cells were washed in cold PBS and lysed in 2 ml/dish cold 1% (wt/vol) Triton X-100, 1 mM CaCl_2_, 1 mM MgCl_2_, 1 mM MnCl_2_, 150 mM NaCl, 5% (wt/vol) glycerol, 50 mM HEPES (pH 7.5) supplemented with protease inhibitor cOmplete and 1 mM PMSF. Cleared lysate was added to MAb VIA4 matrix and HA matrix (Roche) and incubated on head-over shaker for 4 h at 4°C. Beads were washed four times with cold lysis buffer. Proteins were eluted by boiling in nonreducing SDS-PAGE sample buffer, followed by centrifugation. Supernatants were harvested, and 100 mM DTT was added, followed by boiling prior to loading on the gel. Enrichment of DG by wheat germ agglutinin (WGA) affinity purification was performed as reported previously (89).

### Immunoblotting.

Proteins were separated by SDS-PAGE and transferred to nitrocellulose. After blocking in 5% (wt/vol) skim milk in PBS, membranes were incubated with 1 to 10 µg/ml primary antibody in 5% (wt/vol) skim milk and PBS overnight at 4°C. After several washes in PBS, 0.1% (wt/vol) Tween 20 (PBST), secondary antibodies coupled to HRP were diluted 1:5,000 in PBST and applied for 1 h at room temperature. For detection of mouse MAb IgG in IPs using mouse IgG, HRP-conjugated mouse TrueBlot ULTRA secondary antibody was used (Rockland Inc.). After washing in PBS with 280 mM NaCl and 0.2% (wt/vol) Tween 20, blots were developed by enhanced chemiluminescence (ECL) using LiteABlot kit (EuroClone). Signals were acquired by ImageQuant LAS 4000Mini (GE Healthcare Lifesciences). Quantification of Western blots was performed with ImageQuant TL (GE Healthcare Lifesciences).

### RNA interference and CRISPR/Cas9.

RNAi was performed using a custom-made validated small interfering RNA (siRNA) library (Table S2) as described previously (90). For the generation of DG-deficient A549 cells, lentiviral vectors expressing small hairpin RNA (shRNA) were generated and applied as described previously (30). For the generation of LAMP1-deficient A549 cells, CRISPR/Cas9 was applied. Two guide RNAs (gRNAs) targeting the second exon of LAMP-1 were selected using http://crispr.mit.edu/. Primers for both strands covering the two cleavage sites (1F, 5′-**CACCG**CTGCTGACGCACAATGCATG-3′; 1R, 5′-**AAAC**CATGCATTGTGCGTCAGCAG**C**-3′; 2F, 5′-**CACCG**CAACGGGACCGCGTGCATAA-3′; 2R, 5′-**AAAC**TTATGCACGCGGTCCCGTTG**C**-3′ were annealed to make a double-stranded oligonucleotide that was cloned into the Esp3I site of a lentiCRISPR v2 (Addgene, 52961, provided by Christian Widmann, University of Lausanne). Correct insertion was confirmed by sequencing. For generation of lentiviral vectors, each gRNA cloned into lentiCRISPR v2 was cotransfected with psPAX2 and pMD2.G into HEK293T cells using Lipofectamine. Lentiviruses were collected and used to infect A549 cells for genome editing. After 5 days of puromycin selection, single cell clones were obtained by limiting dilution.

10.1128/mBio.02869-18.3 TABLE S2 siRNAs used in the study. Download Table S2, XLSX file, 0.01 MB. Copyright © 2019 Herrador et al. 2019 Herrador et al. This content is distributed under the terms of the Creative Commons Attribution 4.0 International license.

### Virus infections.

Cells were seeded in 96-well plates at 2 × 10^4^ cells per well and grown into confluent monolayers for 16 to 20 h, unless stated otherwise. Cells were treated with drugs as detailed in the specific experiments, followed by infection with the indicated viruses at the defined multiplicity of infection (MOI) for 1 h at 37°C. Unbound virus was removed, cells were washed twice with DMEM, and fresh medium was added. Infection of rLCMV-LASVGP and rLCMV-VSVG were quantified by detection of LCMV NP in immunofluorescence assay (IFA) with MAb 113 as described previously (37). AdV5-GFP was detected via the GFP reporter using direct fluorescence. Perturbation with antibodies to Axl, TIM-1, and functional α-DG was performed as reported previously (30). Infectious virus titers of rLCMV-LASVGP and rLCMV-VSVG were determined by immunofocus assay (IFA) as described previously (91). For AdV gene transfer, monolayers of A549 cells cultured in 10-cm dishes were infected with recombinant human AdV5 expressing α-, β-, γ-, and δ-sarcoglycan and sarcospan at an MOI of 20 PFU/cell for each virus in 10 ml of complete medium overnight at 37°C and 5% CO_2_. The next day, cells were washed twice in serum-free medium, and 20 ml of fresh complete medium was added for another 48 h prior to experiments.

### Virus internalization assays.

For a detailed description of virus internalization assays, see Text S1 in the supplemental material.
