# Supplementary material for: Dynamic Dystroglycan Complexes Mediate Cell Entry of Lassa Virus
Source: mBio. 2019 Mar 26;10(2):e02869-18. doi: 10.1128/mBio.02869-18 (PMC6437060; doi:10.1128/mBio.02869-18)
Supplement: TEXT S1 [file mBio.02869-18-s0001.docx]

**SUPPLEMENTARY INFORMATION**

**Supplementary Materials and Methods**

**Proteomic analysis**

***Sample preparation:*** The AE-MS protocol was modified from (1, 2). MAb VIA4 was conjugated to protein A/G Dynabeads (Dynabead coupling kit, Life Technologies). The VIA4 matrix was washed with lysis buffer, 50 mM Hepes, pH 7.4, 150 mM NaCl, 10% (wt/vol) glycerol, 1 % (wt/vol) NP-40, 1 mM CaCl_2 ,_ cOmplete protease inhibitors (Roche), 1mM PMSF, Phosphostop (Roche) and 5 mM N-ethyl maleimide (NEM), supplemented with 0.2 % (wt/vol) SDS and two times with lysis buffer only. Matrix was then blocked with lysis buffer containing 0.1 % (wt/vol) BSA (Sigma) followed by two washes with lysis buffer. As control, HA matrix (mAb HA7) Dynabeads were used. A549 cells were cultured in three biological replicates (10 x 10 cm dishes, 4 x 10^6^ cells/dish). Cells were washed twice with PBS, 5 ml cold PBS added, cells scraped off and transferred into a 15 ml tube. Cells of 10 dishes were pelleted for 5 min at 300 g at 4°C, suspendend in 800 µl of cold lysis buffer supplemented with 1 mM DTSSP, and reacted for 2 h in the cold. After quenching with 50 mM Tris/HCl, pH 7.5 for 15 min, lysates were centrifuged at 12,000 g for 10 min at 4°C. Lysates were then pre-cleared by incubation with 2 mg of blocked HA Dynabeads for 1 h at 4°C. Lysates were split 50:50 into 2 tubes containing 10 µl VIA4 or HA matrix, followed by affinity enrichment on a head-over shaker for 1 h at 4°C. The matrix was washed three times with complete lysis buffer and once with lysis buffer without detergent. Proteins were eluted by boiling in 30 µl non-reducing SDS-PAGE sample buffer for 5 min, followed by separation of the matrix by centrifugation. Supernatants were harvested and 100 mM DTT added, followed by boiling for 5 min prior to loading on the gel. Samples were split and 10% used for silver staining, 5% for Western blot to verify the specificity of the affinity enrichment, and 85% used for subsequent LC-MS/MS analysis.

***LC-MS/MS analysis:*** Proteins were separated by 1D-SDS-PAGE on two-layer gels (7% and 12% acrylamide) over a distance of 3.0 cm. After rapid Coomassie Blue staining, entire gel lanes were excised into 6 equal regions from top to bottom and in-gel digested with trypsin (Promega) as described (3) and shown in Fig. S2. Data-dependent LC-MS/MS analysis of extracted peptide mixtures after digestion was carried out on a Fusion tri-hybrid orbitrap mass spectrometer (Thermo Fisher Scientific) interfaced to a Dionex RSLC 3000 nano-HPLC. Peptides were separated on a 60 min gradient from 5% to 50% acetonitrile in 0.1% formic acid at 0.3 ul/min on a PepMap column (75 μm ID x 25 cm, 2.0 μm, 100Åµ, Dionex). Full MS survey scans were performed at 120, 000 resolution. In data-dependent acquisition controlled by Xcalibur 2.1 software (Thermo Fisher), the twenty most intense multiply charged precursor ions detected in the full MS survey scan were selected for collision-induced dissociation and analysis in the linear trap with an isolation window of 1.6 m/z and then dynamically excluded from further selection during 60 s. Collections of tandem mass spectra from all five fractions for each sample were pooled for database searching using Mascot (Matrix Science, London, UK; version 2.5.0) against the release 2014_08 of the SWISSPROT database restricted to human taxonomy (20540 sequences). Mass tolerances used were 10 ppm for the precursors and 0.5 Da for CID fragments. The software Scaffold 4.4.1.1 (Proteome Software Inc.) was used to validate MS/MS based peptide identifications (minimum 90% probability) (4) and protein (min 95% probability with 2 peptides minimum) (5), perform dataset alignment and subtraction as well as parsimony analysis to discriminate homologous hits.

***Data processing and statistical analysis:*** Exclusive spectrum counts were further analyzed using the software Perseus 1.5.1.6 (6). Data were filtered for proteins with a minimum of three identifications in at least one group, i.e. either in the three VIA4 or in the three HA samples. Missing values were replaced by a spectral count of 1 and protein abundance plotted logarithmically using GraphPad Prism 5. For statistical analysis, data was log2 transformed. We then conducted a nonparametric two samples test correcting for multiple hypothesis testing and identified 33 proteins significantly enriched in DG AE from A549 cells (FDR ≤0.05, s0=1; s0: the minimal log_10_ fold change). Among the significant hits was the bait DG with 32 fold enrichment in VIA4 versus HA samples (FDR=3.6x10E-7). Median protein abundance differences, measured as log2 spectral count, and p values were plotted using GraphPad Prism 5. The significant protein interactions from this publication have been submitted to the IMEx (http://www.imexconsortium.org) consortium through IntAct and assigned the identifier IM-26486.

**Virus internalization assays**

Purified rLCMV-LASVGP was diluted in PBS and labeled with the thiol-cleavable reagent NHS-SS-biotin (Pierce) as reported (7). The cleavage of the biotin label was verified by reaction with the membrane-impermeable reducing agent Tris(2-carboxyethyl)phosphine (TCEP) (10 mM) (Pierce) for 30 min, which resulted in a loss of >95% of the biotin label. The classical virus internalization assay was performed as described previously (8). Briefly, 4 x 10^5^ A549 cells were seeded per well of M6 tissue culture plates and cultured for 16-24 h to obtain >90% closed monolayers. Medium was removed and cells washed twice with cold HBSS and chilled on ice for 5 min. Cold solution containing NHS-SS-biotinylated rLCMV-LASVGP (50 PFU/cell) in HBSS was added. After incubation for 1 h on ice, unbound virus was removed and cells were washed with cold HBSS. For internalization, cells were rapidly shifted to 37°C by adding pre-warmed complete medium. After the indicated incubation times, medium was removed and cells chilled on ice. TCEP (15 mM) in 50 mM HEPES, pH 7.5, 150 mM NaCl, 1 mM CaCl_2_, 1 mM MgCl_2_ was added (1 ml/well) and applied twice for 15 min on ice. Cells were washed three times with cold HBSS, and remaining TCEP quenched with 2 ml/well 100 mM iodoacetamide in HBSS for two times 10 min, followed by cell lysis using 1% (wt/vol) Triton X-100, 0.1% (wt/vol) SDS, 50 mM Tris-HCl, pH 7.5, 150 mM NaCl, 1 mM EDTA, 1 mM PMSF, and protease inhibitors cOmplete, Roche. To cleared lysates, 10^7^ PFU/ml purified, unlabeled rLCMV-LASVGP was added as carrier. LCMV GP2 was isolated by IP using mAb 83.6 anti-LCMV GP2 immobilized on Sepharose 4B as described (8). Immunocomplexes were separated by nonreducing SDS-PAGE. Biotinylated LCMV GP2 was detected by Western blotting with HRP-conjugated streptavidin using ECL for detection.

To monitor virus-induced receptor uptake, A549 cells were cultured in M6 plates as described above (duplicate wells per specimen) and subjected to cell surface biotinylation with 1ml/well of 1 mM of the thiol-cleavable reagent EZ-Link™-Sulfo-NHS-SS-biotin (Thermo scientific) in cold HBSS for two times 20 min at 4°C. Reaction was quenched with 2 ml/well of cold 50 mM glycine, 150 mM NaCl, 1 mM MgCl_2_, 0.1 mM CaCl_2_, pH 8.0. Cells were washed four times with cold HBSS and subjected to virus internalization assay using NHS-SS-biotinylated rLCMV-LASVGP (50 PFU/cell) in HBSS, as described above. As control, a “mock virus preparation” was prepared from conditioned supernatant of uninfected BHK21 cells subjected to PEG precipitation and purification over a renografin gradient analogous to the virus preparation as described (9). Surface biotinylated cells were incubated with virus and mock preparation for 2 h on ice to allow attachment without internalization. After three washes with cold HBSS, cells were rapidly shifted to 37°C by adding pre-warmed complete medium. At the indicated incubation times, medium was removed and cells chilled on ice. TCEP (15 mM) in 50 mM HEPES, pH 7.5, 150 mM NaCl, 1 mM CaCl_2_, 1 mM MgCl_2_ was added (1 ml/well) and applied twice for 15 min on ice. Cells were washed three times with cold HBSS, and remaining TCEP quenched with 2 ml/well 100 mM iodoacetamide in HBSS for two times 10 min. After three washes with copious volumes of HBSS, cells were lysed as described above. Cleared lysats were split 50:50. One half of the lysate was subjected to IP with mAb 83.6 to LASV GP as detailed above and the other half to IP with VI4A matrix to glycosylated α-DG. Immunocomplexes were washed four times with lysis buffer, once with lysis buffer without detergent and eluted by boiling with non-reducing SDS-PAGE sample buffer. Eluted proteins were separated by nonreducing SDS-PAGE and biotinylated LCMV GP2 and α-DG detected by Western blotting with HRP-conjugated streptavidin using ECL for detection.

**REFERENCES**

1. Schneider C, Newman RA, Sutherland DR, Asser U, Greaves MF. 1982. A one-step purification of membrane proteins using a high efficiency immunomatrix. J Biol Chem 257:10766-9.

2. Gerold G, Meissner F, Bruening J, Welsch K, Perin PM, Baumert TF, Vondran FW, Kaderali L, Marcotrigiano J, Khan AG, Mann M, Rice CM, Pietschmann T. 2015. Quantitative Proteomics Identifies Serum Response Factor Binding Protein 1 as a Host Factor for Hepatitis C Virus Entry. Cell Rep 12:864-78.

3. Wilm M, Shevchenko A, Houthaeve T, Breit S, Schweigerer L, Fotsis T, Mann M. 1996. Femtomole sequencing of proteins from polyacrylamide gels by nano-electrospray mass spectrometry. Nature 379:466-9.

4. Keller A, Nesvizhskii AI, Kolker E, Aebersold R. 2002. Empirical statistical model to estimate the accuracy of peptide identifications made by MS/MS and database search. Anal Chem 74:5383-92.

5. Cox J, Neuhauser N, Michalski A, Scheltema RA, Olsen JV, Mann M. 2011. Andromeda: a peptide search engine integrated into the MaxQuant environment. J Proteome Res 10:1794-805.

6. Cox J, Mann M. 2012. 1D and 2D annotation enrichment: a statistical method integrating quantitative proteomics with complementary high-throughput data. BMC Bioinformatics 13 Suppl 16:S12.

7. Moraz ML, Pythoud C, Turk R, Rothenberger S, Pasquato A, Campbell KP, Kunz S. 2013. Cell entry of Lassa virus induces tyrosine phosphorylation of dystroglycan. Cell Microbiol 15:689-700.

8. Rojek JM, Perez M, Kunz S. 2008. Cellular entry of lymphocytic choriomeningitis virus. J Virol 82:1505-17.

9. Fedeli C, Torriani G, Galan-Navarro C, Moraz ML, Moreno H, Gerold G, Kunz S. 2018. Axl Can Serve as Entry Factor for Lassa Virus Depending on the Functional Glycosylation of Dystroglycan. J Virol 92.
